# Health-Related Quality of Life and Family Impact in Young Children with Infantile Epileptic Spasms Syndrome: A Cross-Sectional Caregiver-Reported Study

**DOI:** 10.3390/jcm15197706

**Published:** 2026-10-05

**Authors:** Natalia Kasaraba, Anna Rozensztrauch

**Affiliations:** Department of Pediatrics and Coordinated Child Care, Wroclaw Medical University, 50-367 Wroclaw, Poland; natalia.kasaraba2001@gmail.com

**Keywords:** infantile epileptic spasms syndrome, West syndrome, health-related quality of life, family impact, clinical comorbidities, epilepsy, caregivers

## Abstract

**Background/Objectives:** Infantile epileptic spasms syndrome (IESS) is a developmental and epileptic encephalopathy characterized by epileptic spasms and epileptiform EEG abnormalities. Classical West syndrome represents a phenotype within the IESS spectrum characterized by epileptic spasms, hypsarrhythmia, and developmental plateau or regression. Beyond seizure control, assessment of health-related quality of life (HRQoL) and family impact may provide important information on the long-term burden of the disease. This study aimed to characterize caregiver-reported HRQoL in young children with IESS and family impact and to explore clinical and perinatal factors associated with these outcomes. **Methods:** This cross-sectional caregiver-proxy study included 60 parents or primary caregivers of children aged 1 month to 4 years who had previously received a physician-established diagnosis of West syndrome; in the present manuscript, the cohort is described within the broader contemporary IESS framework. Children’s HRQoL was assessed using age-appropriate versions of the Pediatric Quality of Life Inventory (PedsQL), while family impact was assessed using the PedsQL Family Impact Module (FIM). Parametric and non-parametric statistical methods were applied as appropriate. All analyses were exploratory, and no adjustment for multiple comparisons or multivariable adjustment for potential confounders was performed. **Results:** The mean PedsQL Total Scale Score was 64.53 ± 19.35 among children aged 1–24 months assessed with the PedsQL Infant Scales and 56.34 ± 23.29 among children aged 2–4 years assessed with the PedsQL 4.0 Generic Core Scales. In exploratory pooled analyses, children with additional congenital, genetic, structural, or systemic conditions had lower age-appropriate Total Scale Scores than children without such conditions (52.93 ± 21.55 vs. 64.40 ± 22.08; unadjusted mean difference −11.48 points, 95% CI −22.75 to −0.20; nominal *p* = 0.046). Physical health scores were also lower in children with additional conditions (53.78 ± 27.62 vs. 69.60 ± 26.71; nominal *p* = 0.012). In the sensitivity analysis restricted to children aged 2–4 years assessed with a single PedsQL instrument, the Total Scale Score difference was similar in direction but did not reach the nominal significance threshold (48.76 ± 20.86 vs. 62.35 ± 23.76; mean difference −13.59 points, 95% CI −27.57 to 0.38; nominal *p* = 0.056). Within the FIM, the lowest mean scores were observed for Worry (24.17 ± 21.20) and Daily Activities (35.00 ± 28.57). **Conclusions:** Caregiver-reported HRQoL and family impact varied substantially among young children with IESS and their families. Additional congenital, genetic, structural, or systemic conditions were associated with lower scores in selected child HRQoL domains, although these exploratory unadjusted findings may be affected by residual confounding. The findings support the inclusion of child HRQoL and family impact measures alongside neurological outcomes in the follow-up of children with IESS.

## 1. Introduction

Advances in neonatal diagnosis and management have improved the survival of infants with severe neurological disorders and other conditions requiring specialized neonatal care. As survival has improved, increasing attention has been directed toward long-term neurodevelopmental outcomes, functional status, and health-related quality of life (HRQoL). Infantile epileptic spasms syndrome (IESS) is a developmental and epileptic encephalopathy characterized by the onset of epileptic spasms, typically between 1 and 24 months of age, together with epileptiform EEG abnormalities. Hypsarrhythmia and developmental plateau or regression are frequently observed but are not required for the diagnosis of IESS. Classical West syndrome represents a phenotype within the IESS spectrum characterized by the triad of epileptic spasms, hypsarrhythmia, and developmental plateau or regression [1,2,3]. Consequently, long-term outcomes have become an important component of the clinical management of these children. IESS usually develops during the first year of life and has a heterogeneous clinical course influenced by the underlying etiology. Although early diagnosis and treatment improve seizure control, many children continue to present with epilepsy, motor impairment, cognitive deficits, communication disorders, visual impairment, and other neurological comorbidities [3,4,5,6]. Therefore, long-term outcomes should be assessed not only by seizure control but also by functional status and HRQoL.

HRQoL has become an important outcome measure in children with chronic neurological disorders because it reflects physical, emotional, social, and cognitive functioning in everyday life. Patient-reported outcomes are increasingly recognized as an important component of long-term follow-up in children with chronic diseases. Previous studies have demonstrated impaired HRQoL in children with epilepsy and other neurological disorders [7,8]. However, studies evaluating HRQoL specifically in children with West syndrome remain limited, particularly those investigating factors associated with poorer outcomes [5,6]. Long-term follow-up studies indicate that neurological sequelae, developmental impairment, and associated comorbidities continue to affect children’s functioning even after successful seizure control [5,6]. The consequences of West syndrome extend beyond the affected child and may influence the entire family. Parents frequently report increased stress, anxiety, financial burden, and limitations in daily activities related to long-term caregiving [9,10,11]. These factors may reduce parental QoL and family functioning [9,10,11]. Therefore, the aim of this study was to characterize caregiver-reported HRQoL in young children with IESS and family impact and to explore demographic, perinatal, and clinical factors associated with these outcomes. Therefore, the aim of this study was to characterize caregiver-reported HRQoL in young children with IESS and family impact and to explore demographic, perinatal, and clinical factors associated with these outcomes.

## 2. Materials and Methods

### 2.1. Study Design and Participants

This cross-sectional caregiver-proxy study was conducted at a tertiary referral center for pediatric neurology in Wroclaw, Poland. Participants were recruited consecutively during routine follow-up visits at a tertiary referral center for pediatric neurology in Wroclaw, Poland, between February and May 2026. Eligible parents or primary caregivers were invited to participate during the predefined recruitment period. The final study sample comprised 60 caregivers of children meeting the eligibility criteria. A formal screening log documenting the total number of potentially eligible families approached, refusals, and pre-enrolment exclusions was not available; therefore, these numbers could not be reconstructed reliably.

At the time of clinical diagnosis, West syndrome had been diagnosed by a pediatric neurologist as part of routine clinical care. For the purposes of the present manuscript, terminology was harmonized with the current International League Against Epilepsy (ILAE) classification, and the study population is described within the broader framework of infantile epileptic spasms syndrome (IESS). Classical West syndrome represents a phenotype within the IESS spectrum characterized by the triad of epileptic spasms, hypsarrhythmia or modified hypsarrhythmia, and developmental plateau or regression. Because the available study dataset did not permit independent retrospective verification of all components of the classical West syndrome triad in every participant, individual patients were not retrospectively reclassified according to the presence or absence of the complete triad.

The study included 60 parents or primary caregivers of children aged 1 month to 4 years at the time of HRQoL assessment. The reported age range refers to the child’s age at the time of HRQoL assessment and not to the age at onset of epileptic spasms. The inclusion criteria were: (1) a previously physician-established diagnosis of West syndrome; (2) child age between 1 month and 4 years at the time of HRQoL assessment; and (3) informed consent provided by the parent or legal guardian. No formal a priori sample-size calculation was performed. The study size reflected the number of eligible participants recruited during the predefined data-collection period. Study questionnaires were administered in Polish. Authorized Polish-language versions of the PedsQL instruments were used. The author-developed demographic and clinical questionnaire was originally prepared and administered in Polish. Missing PedsQL items were handled according to the instrument scoring guidelines. Scale scores were not calculated when more than 50% of the items within a scale were missing. When at least 50% of the items were completed, the score was calculated from the available completed items according to the standard PedsQL scoring procedure. Participants were excluded from a specific analysis only when the outcome required for that analysis could not be calculated. Thus, available data were retained for other applicable analyses whenever possible.

### 2.2. Demographic and Clinical Characteristics

Clinical and demographic data were collected using an author-developed questionnaire and medical information provided by caregivers. The collected variables included the child’s age, sex, gestational age at birth, mode of delivery, additional congenital, genetic, structural, or systemic conditions, and selected clinical characteristics. Information on caregiver age, education, place of residence, and marital status was also collected. Standardized diagnostic criteria and severity measures were not systematically available for all reported conditions; therefore, they were analysed as reported clinical characteristics rather than as independently adjudicated diagnoses. Selected clinical characteristics included caregiver-provided epilepsy-related information, visual impairment, speech disorders, gastrointestinal motility disorders, hypotonia, feeding difficulties, hypoxic–ischemic encephalopathy, recurrent infections requiring antibiotic therapy, poor weight gain, and other reported comorbidities. Epilepsy-related information was treated as non-standardized caregiver-provided clinical information. The available source information did not include a standardized standalone epilepsy-status item with predefined response options or a defined reference period, nor standardized data on seizure type, seizure frequency, seizure-free interval, EEG response, relapse, treatment response, or medication burden. Accordingly, this information was not interpreted as an objective measure of current epilepsy status, seizure control, epilepsy severity, or treatment resistance. Variables allowing multiple responses were analysed separately. Additional reported congenital, genetic, structural, or systemic conditions were analysed as a binary variable (present/absent).

### 2.3. Assessment of Children’s Health-Related Quality of Life

Children’s health-related quality of life (HRQoL) was assessed using age-appropriate parent-proxy versions of the Pediatric Quality of Life Inventory (PedsQL). The PedsQL Infant Scales were used for children aged 1–12 months and 13–24 months [12,13], whereas the PedsQL 4.0 Generic Core Scales were used for children aged 2–4 years [14,15,16,17]. Although the age-specific instruments differ in item and domain composition, they use the same response format and scoring principle, with item responses reverse-scored and linearly transformed to a 0–100 scale; higher scores indicate better HRQoL. Total Scale Scores were calculated from the applicable items within each age-specific instrument. For descriptive age-specific reporting, children aged 1–12 months (*n* = 5) and 13–24 months (*n* = 12) were combined into an Infant Scale group aged 1–24 months (*n* = 17) because of the small number of participants in the youngest subgroup. Cognitive Functioning and School/Daycare Functioning were analysed only within the age groups for which the respective domains were applicable. Because the PedsQL Infant Scales and the PedsQL 4.0 Generic Core Scales differ in item and domain composition, scores derived from these instruments were not assumed to be psychometrically equivalent despite sharing a common 0–100 metric. Age-specific Total Scale Scores were therefore reported separately. Pooled full-cohort analyses were retained only as exploratory analyses. In addition, a sensitivity analysis was performed in the larger subgroup of children aged 2–4 years assessed exclusively with the PedsQL 4.0 Generic Core Scales (*n* = 43). Owing to the small size of the Infant Scales subgroup (*n* = 17), results in this subgroup were interpreted primarily descriptively.

### 2.4. Assessment of Family Impact

Family impact was assessed using the PedsQL 2.0 Family Impact Module (FIM), a 36-item caregiver-reported instrument comprising eight domains: Physical Functioning, Emotional Functioning, Social Functioning, Cognitive Functioning, Communication, Worry, Daily Activities, and Family Relationships. Item responses were reverse-scored and transformed to a 0–100 scale, with higher scores indicating better functioning. In accordance with the standard scoring procedure, three summary scores were calculated: the Total Impact Score, based on all 36 items; the Parent HRQoL Summary Score, based on the Physical, Emotional, Social, and Cognitive Functioning domains; and the Family Functioning Summary Score, based on the Daily Activities and Family Relationships domains. Individual domain scores were also calculated and reported separately [18].

### 2.5. Statistical Analysis

Statistical analyses were performed using IBM SPSS Statistics for Windows, version 29.0 (IBM Corp., Armonk, NY, USA).

Quantitative variables were presented as mean and standard deviation (SD) or median with minimum and maximum values, depending on data distribution. Categorical variables were expressed as numbers and percentages. The normality of data distribution was assessed using the Shapiro–Wilk test. Comparisons between two independent groups were performed using Student’s *t*-test or the Mann–Whitney U test, as appropriate. Comparisons among more than two groups were conducted using one-way analysis of variance (ANOVA) or the Kruskal–Wallis test. Correlations between quantitative variables were evaluated using Spearman’s rank correlation coefficient. The chi-square test or Fisher’s exact test was used for categorical variables, depending on the expected cell frequencies. Because of the limited sample size, selected variables were combined before statistical analysis. Child age was categorized as 1–24 months and 2–4 years. Gestational age was categorized as 22–37 weeks, 38–40 weeks, and >40 weeks. Additional congenital, genetic, structural, or systemic conditions were analysed as a binary variable (present/absent). All statistical analyses were considered exploratory. Two-sided *p*-values < 0.05 were regarded as nominally statistically significant. For the instrument-specific sensitivity analysis, comparisons between children with and without additional congenital, genetic, structural, or systemic conditions were performed within the 2–4-year subgroup only. Student’s *t*-test or the Mann–Whitney U test was used as appropriate according to the distribution of the analysed outcome. These subgroup analyses were exploratory and were not adjusted for multiple comparisons. No adjustment for multiple comparisons was applied; therefore, all reported *p*-values should be interpreted as nominal and the findings as hypothesis-generating rather than confirmatory. Between-group comparisons are reported with the number of participants contributing to each group, group-specific descriptive statistics, the estimated unadjusted between-group difference with its 95% confidence interval, and the numerical two-sided nominal *p*-value. Because of the limited sample size, no multivariable adjustment for potential confounders was performed. Consequently, these exploratory associations may be affected by residual confounding and should not be interpreted as independent effects.

## 3. Results

### 3.1. Demographic and Clinical Characteristics

The study included 60 children in the IESS cohort and their primary caregivers. As described in the Methods, all children had a previously physician-established diagnosis of West syndrome, while the broader contemporary IESS terminology is used throughout the revised manuscript. Among caregivers, 54 (90.0%) were mothers and 6 (10.0%) were fathers, with caregiver age ranging from 19 to 55 years. Thirty-five caregivers (58.3%) had higher education, and 47 (78.3%) were married. The study population included 32 boys (53.3%) and 28 girls (46.7%). Five children (8.3%) were aged 1–12 months, 12 (20.0%) were aged 13–24 months, and 43 (71.7%) were aged 2–4 years at the time of HRQoL assessment.

Fourteen children (23.3%) were born preterm: 3 (5.0%) at 22–28 weeks of gestation, 5 (8.3%) at 29–32 weeks, and 6 (10.0%) at 33–37 weeks. Thirty-two children (53.3%) were born at 38–40 weeks of gestation, and 14 (23.3%) after 40 completed weeks.

Additional congenital, genetic, structural, or systemic conditions were reported in 30 children (50.0%). These included hydrocephalus in 6 children (10.0%), trisomy 21 in 5 (8.3%), congenital heart defects in 4 (6.7%), and single cases of anal atresia, renal hypoplasia, genital developmental abnormalities, and vertebral anomalies. Other reported conditions included STXBP1 and HCN1 variants, chromosome 15q11–q13 microduplication syndrome, DiGeorge syndrome, neurofibromatosis type 1, and polymicrogyria. Because multiple conditions could be reported in the same child, individual categories were not mutually exclusive. Given their clinical heterogeneity, these conditions were pooled only for exploratory analyses and were not considered a uniform etiological category of IESS.

At least one predefined clinical condition was reported in 49 children (81.7%). The most frequently reported were hypotonia in 29 children (48.3%), recurrent infections requiring antibiotic therapy in 27 (45.0%), feeding difficulties in 15 (25.0%), a history of hypoxic–ischemic encephalopathy in 13 (21.7%), and poor weight gain in 12 (20.0%).

### 3.2. Children’s Health-Related Quality of Life and Associated Factors

Children’s HRQoL was assessed using age-appropriate PedsQL instruments. The pooled full-cohort Total Scale Score was 58.67 ± 22.39 and is presented as a descriptive summary of age-appropriate PedsQL scores. To account for differences in item and domain composition between the age-specific instruments, Total Scale Scores are also reported separately according to the instrument used. Among children aged 1–24 months assessed with the PedsQL Infant Scales (*n* = 17), the mean Total Scale Score was 64.53 ± 19.35. Among children aged 2–4 years assessed with the PedsQL 4.0 Generic Core Scales (*n* = 43), the mean Total Scale Score was 56.34 ± 23.29.

Among children aged 2–4 years assessed with the PedsQL 4.0 Generic Core Scales (*n* = 43), the mean Total Scale Score was 53.96 ± 23.00. In the full cohort, the descriptive physical health, Emotional Functioning, and Social Functioning scores were 61.69 ± 28.09, 60.67 ± 23.61, and 58.08 ± 28.70, respectively. Cognitive Functioning was assessed only in children aged 1–24 months (*n* = 17; 43.75 ± 34.49), whereas School/Daycare Functioning could be calculated for 29 children aged 2–4 years with sufficient completed items (*n* = 29; 52.30 ± 29.54) (Table 1).

In exploratory pooled analyses, gestational age category was not associated with the pooled Total Scale Score (22–37 weeks: 51.74 ± 20.95; 38–40 weeks: 60.70 ± 21.57; >40 weeks: 60.94 ± 25.68; F(2,57) = 0.87; nominal *p* = 0.424). No statistically significant associations were observed for physical health, Emotional Functioning, Social Functioning, or Cognitive Functioning scores (all nominal *p* > 0.05). School/Daycare Functioning, calculated for 29 children with sufficient completed items, differed across gestational age categories (22–37 weeks: *n* = 5, 30.00 ± 19.18; 38–40 weeks: *n* = 15, 48.89 ± 30.19; >40 weeks: *n* = 9, 70.37 ± 23.98; F(2,26) = 3.87; nominal *p* = 0.034). Given the small subgroup sizes, incomplete School/Daycare data, and the absence of adjustment for multiple comparisons, this finding should be interpreted as exploratory and hypothesis-generating.

Children with additional congenital, genetic, structural, or systemic conditions had lower pooled Total Scale Scores than children without such conditions (52.93 ± 21.55 vs. 64.40 ± 22.08; mean difference −11.48, 95% CI −22.75 to −0.20; nominal *p* = 0.046). Physical health scores were also lower in children with additional conditions (53.78 ± 27.62 vs. 69.60 ± 26.71; mean difference −15.81, 95% CI −29.86 to −1.77; nominal *p* = 0.012). No statistically significant between-group differences were identified for Emotional Functioning, Social Functioning, Cognitive Functioning, or School/Daycare Functioning (Table 2). These comparisons were exploratory and unadjusted and may therefore be affected by residual confounding. Complete results of the exploratory analyses, including non-significant findings, are provided in Supplementary Table S1.

To assess whether the observed association remained evident when a single age-specific instrument was used, a sensitivity analysis was performed in children aged 2–4 years assessed exclusively with the PedsQL 4.0 Generic Core Scales (*n* = 43). Nineteen children had additional congenital, genetic, structural, or systemic conditions and 24 did not. The Total Scale Score was lower among children with additional conditions (48.76 ± 20.86 vs. 62.35 ± 23.76), corresponding to an unadjusted mean difference of −13.59 points (95% CI −27.57 to 0.38; nominal *p* = 0.056). Physical Functioning was also lower among children with additional conditions (46.88 ± 29.44 vs. 67.97 ± 29.49; nominal *p* = 0.016), as was Social Functioning (44.21 ± 18.80 vs. 60.21 ± 24.91; nominal *p* = 0.013). Emotional Functioning did not differ between groups (57.11 ± 25.46 vs. 59.58 ± 23.86; nominal *p* = 0.744). School/Daycare Functioning did not differ significantly among children with available data (41.67 ± 28.17 vs. 56.35 ± 29.69; nominal *p* = 0.238). These sensitivity analyses were exploratory and unadjusted and were not adjusted for multiple comparisons.

### 3.3. Clinical Factors Associated with Family Impact

Family impact was assessed using the PedsQL Family Impact Module. The mean Total Impact Score was 47.78 ± 22.00, the Parent HRQoL Summary Score was 52.25 ± 25.24, and the Family Functioning Summary Score was 52.14 ± 26.16. Among the individual domains, the lowest mean scores were observed for Worry (24.17 ± 21.20) and Daily Activities (35.00 ± 28.57). Descriptive results for the summary scores and individual FIM domains are presented in Table 3.

In exploratory unadjusted analyses, caregivers of children with at least one reported clinical condition (*n* = 49) had a lower mean FIM Total Impact Score than caregivers of children with no reported clinical condition (*n* = 11; 45.52 ± 21.77 vs. 57.83 ± 21.10; mean difference −12.31, 95% CI −26.77 to 2.16; nominal *p* = 0.094). The Parent HRQoL Summary Score was also lower in the group with at least one reported clinical condition (49.67 ± 25.54 vs. 63.75 ± 21.23; mean difference −14.08, 95% CI −30.68 to 2.51; nominal *p* = 0.095). Family Functioning Summary Scores did not differ between groups (median 56.25 vs. 59.38; nominal *p* = 0.833). None of these comparisons reached the nominal *p* < 0.05 threshold. These findings were exploratory, unadjusted, and may be affected by residual confounding (Table 4; Supplementary Table S2).

## 4. Discussion

The present study provides a caregiver-reported perspective on HRQoL and family impact in young children with IESS. Mean PedsQL Total Scale Scores were 64.53 in children aged 1–24 months and 56.34 in those aged 2–4 years; these age-group results were interpreted separately because different age-appropriate PedsQL instruments were used. In exploratory pooled analyses, additional congenital, genetic, structural, or systemic conditions were associated with lower Total Scale Scores and lower physical health scores. In the sensitivity analysis restricted to children aged 2–4 years assessed with a single PedsQL instrument, the difference in Total Scale Score was similar in direction and magnitude to that observed in the pooled analysis, although the confidence interval included the null value. This finding supports a cautious interpretation of the pooled association and indicates that confirmation in larger instrument-homogeneous cohorts is needed. Family-impact scores showed substantial variability, with the lowest mean scores observed for Worry and Daily Activities. In exploratory unadjusted analyses, FIM summary scores did not differ significantly between children with at least one reported clinical condition and those with none. Because no healthy or disease-control group was included, the HRQoL findings should be interpreted descriptively rather than as evidence of impairment relative to age-matched peers.

Previous studies have also reported reduced HRQoL in children with West syndrome. Wanigasinghe et al. [7] observed lower HRQoL at six years of age, particularly in the physical domain. Differences from the present findings may reflect variation in age, developmental stage, underlying neurological conditions, and the measures used to assess HRQoL. Together, these observations support the relevance of HRQoL as an important outcome alongside conventional neurological and epilepsy-related measures.

The multidimensional nature of HRQoL in West syndrome has also been highlighted by Bhanudeep et al. [8], who reported associations with cognitive and motor impairment, sleep disturbances, behavioral difficulties, and persistent epilepsy. More broadly, studies in childhood epilepsy suggest that HRQoL reflects the combined influence of clinical and psychosocial factors rather than seizure status alone [5,6]. This broader perspective is consistent with the variability observed in our cohort and supports the assessment of everyday functioning and well-being alongside conventional clinical outcomes.

Children with additional congenital, genetic, structural, or systemic conditions had lower pooled PedsQL Total Scale Scores and lower physical health scores. This finding should be interpreted cautiously because the pooled category comprised clinically heterogeneous conditions and was not intended to represent a uniform etiological classification of IESS. Contemporary literature emphasizes the substantial etiological heterogeneity of IESS, including structural, genetic, metabolic, and other causes [2,3,4,19]. Accordingly, the observed association should not be interpreted as evidence of an effect of any specific etiology but may instead reflect greater clinical complexity and associated functional burden. Future studies with larger cohorts and contemporary etiological classification are needed to examine associations between specific etiological groups and HRQoL.

The lowest mean FIM domain scores were observed for Worry and Daily Activities, indicating that concerns about the child’s health and the practical demands of everyday family life may represent particularly important dimensions of caregiver-reported burden. In the exploratory comparison of children with at least one reported clinical condition versus those with none, the Total Impact Score and Parent HRQoL Summary Score were numerically lower in the group with reported clinical conditions, although the confidence intervals included the null value and the differences did not reach the nominal *p* < 0.05 threshold. These findings should therefore be interpreted as descriptive and hypothesis-generating rather than as evidence of independent effects. Previous studies have similarly described psychological, social, and financial burden among families of children with West syndrome and childhood epilepsy [9,10,11]. Together, these observations support the relevance of assessing family outcomes alongside child-centered measures.

The broader pattern of child and family burden is consistent with research in other rare and severe neurological disorders. Johansen et al. [6] reported an association between child QoL and parental life satisfaction, while studies in chronic neurological and neuromuscular disorders have similarly described substantial family burden [6,20].

Treatment-related factors are clinically relevant in IESS and may also be associated with child functioning and family burden. However, the present study was specifically designed to evaluate caregiver-reported child HRQoL and family impact rather than treatment efficacy or comparative therapeutic outcomes. Detailed information on individual treatment regimens, treatment sequence, hormonal therapy, antiseizure medication burden, EEG response, and treatment response was not systematically collected and therefore could not be incorporated into treatment-stratified analyses. Accordingly, the present findings should be interpreted as describing HRQoL and family impact across a clinically heterogeneous IESS cohort rather than outcomes associated with therapeutic approaches. Importantly, the simultaneous assessment of child HRQoL and family impact provides a complementary perspective to conventional seizure- and treatment-focused outcomes and may capture dimensions of disease burden that are not reflected by seizure control alone. Future prospective studies integrating standardized HRQoL and family-impact measures with detailed treatment, seizure, EEG, and neurodevelopmental data are warranted.

### Clinical Implications

The present findings support consideration of HRQoL and family impact alongside conventional neurological outcomes in the clinical follow-up of young children with IESS. Children with additional congenital, genetic, structural, or systemic conditions may require particular attention because these conditions were associated with lower scores in selected child HRQoL domains in exploratory analyses. Assessment of family impact may also help identify areas in which additional multidisciplinary support is needed. Given the exploratory and unadjusted nature of the analyses, these findings should not be used to infer independent effects of individual clinical characteristics.

## 5. Limitations

The present study has several limitations. First, the cross-sectional design does not allow causal relationships or changes in HRQoL and family functioning over time to be determined. Second, the relatively small sample size limits statistical power, generalizability, and the possibility of conducting detailed subgroup analyses. IESS is a relatively uncommon and clinically heterogeneous epilepsy syndrome, which presents practical challenges for recruiting large homogeneous cohorts. Third, HRQoL was assessed by parent or caregiver proxy report. Proxy assessment may not fully reflect the child’s subjective experience; however, it is necessary in infants and young children who are unable to provide reliable self-reports.

The broad age range of the study population represents an additional limitation because developmental expectations differ considerably between infancy and four years of age. Although age-appropriate PedsQL versions were used, these instruments differ in item and domain composition. Age-specific Total Scale Scores were therefore additionally reported separately, while pooled full-cohort analyses were considered exploratory and should not be interpreted as demonstrating psychometric equivalence between the different PedsQL versions. Cognitive Functioning and School/Daycare Functioning were interpreted only within the age groups for which the respective domains were applicable.

The category of additional congenital, genetic, structural, or systemic conditions included clinically heterogeneous disorders and should not be interpreted as an etiological classification of IESS. Detailed etiological classification was not available in the present dataset, preventing analyses according to specific etiological groups. Furthermore, no standardized neurodevelopmental, cognitive, adaptive, motor, or functional severity assessment was available. Consequently, residual confounding by neurodevelopmental severity and broader clinical complexity cannot be excluded.

Detailed treatment-related information, including specific therapies, treatment sequence, seizure response, EEG response, relapse, and medication burden, was not systematically available. Consequently, treatment-specific effects on HRQoL and family impact could not be evaluated, and treatment-related heterogeneity remains a potential source of residual confounding. Epilepsy-related caregiver-provided information was not based on a standardized standalone epilepsy-status item and did not permit a clinically interpretable or reproducible classification of current seizure status, seizure control, or treatment resistance. Accordingly, this information was not used to support conclusions regarding epilepsy severity or treatment response.

In addition, multiple exploratory statistical comparisons were performed without adjustment for multiplicity; therefore, the reported *p*-values should be regarded as nominal and the findings as hypothesis-generating. The study did not include an age-matched healthy control group or a disease-control group of children with other forms of epilepsy. Consequently, the observed PedsQL scores cannot be interpreted as demonstrating impairment relative to healthy peers or other pediatric epilepsy populations. Although an instrument-specific sensitivity analysis was performed in the larger 2–4-year subgroup, the small Infant Scales subgroup limited the possibility of robust instrument-specific inferential analyses in younger children. Consequently, comparisons across the age-specific PedsQL instruments should remain descriptive, and pooled analyses should be regarded as exploratory. Future studies incorporating larger cohorts, standardized neurodevelopmental assessment, detailed clinical and treatment data, and appropriately matched comparator groups are warranted.

## 6. Conclusions

Despite these limitations, the present study provides additional insight into caregiver-reported HRQoL and family impact in infants and young children with IESS. The findings support a multidimensional approach to outcome assessment that considers child HRQoL, associated clinical conditions, and family impact alongside conventional neurological outcomes. The observed associations were exploratory and unadjusted and therefore require confirmation in larger, prospectively characterized cohorts. Future studies incorporating standardized neurodevelopmental assessment, contemporary etiological classification, detailed seizure and treatment data, and longitudinal follow-up are needed to clarify these relationships and their evolution over time.

## Figures and Tables

**Table 1:** Descriptive PedsQL scores for the full cohort and according to the age-specific instrument used.

	*n*	M	SD	Observed Min	Observed Max	Q1	Me	Q3
Pooled Total Scale Score	60	58.67	22.39	6.94	100.00	42.08	63.14	77.38
PF	60	61.69	28.09	0.00	100.00	41.16	68.75	84.38
EF	60	60.67	23.61	5.00	100.00	45.83	64.79	80.00
SF	60	58.08	28.70	0.00	100.00	38.75	57.50	85.00
CF	17	43.75	34.49	0.00	100.00	6.25	38.89	72.22
SCHF	29	52.30	29.54	16.67	100.00	33.33	50.00	75.00
Total Scale Score- Infant Scales, 1–24 months	17	64.53	19.35					
Total Scale Score—Generic Core Scales, 2–4 years	43	56.34	23.29					

M, mean; SD, standard deviation; Q1, first quartile; Me, median; Q3, third quartile; Min., minimum value; Max., maximum value; PF, physical functioning; SF, social functioning; CF, cognitive functioning; SCHF, daycare/preschool/school functioning; EF, emotional functioning. The pooled full-cohort scores are presented descriptively and do not imply psychometric equivalence between the age-specific PedsQL instruments. Age-specific Total Scale Scores are therefore additionally reported separately for children assessed with the PedsQL Infant Scales and the PedsQL 4.0 Generic Core Scales. Cognitive Functioning was applicable only to the Infant Scales group. School/Daycare Functioning was applicable to the Generic Core group and was calculated only for participants with sufficient completed School/Daycare items (*n* = 29). The term “physical health score” is used in the pooled presentation because the composition of the physical construct differs between the age-specific PedsQL versions.

**Table 2:** Exploratory associations between additional congenital, genetic, structural, or systemic conditions and PedsQL scores.

PedsQL Outcome	Conditions Present	Conditions Absent	Difference (95% CI)	*p*
Total Scale Score	*n* = 30; 52.93 ± 21.55	*n* = 30; 64.40 ± 22.08	−11.48 (−22.75 to −0.20)	0.046
Physical health	*n* = 30; 53.78 ± 27.62	*n* = 30; 69.60 ± 26.71	−15.81 (−29.86 to −1.77)	0.012
Emotional Functioning	*n* = 30; 58.67 ± 24.05	*n* = 30; 62.67 ± 23.39	−4.00 (−16.26 to 8.26)	0.516
Social Functioning	*n* = 30; 52.67 ± 30.05	*n* = 30; 63.50 ± 26.69	−10.83 (−25.53 to 3.86)	0.113
Cognitive Functioning	*n* = 11; 39.46 ± 37.60	*n* = 6; 51.62 ± 29.42	−12.16 (−50.12 to 25.79)	0.505
School/Daycare Functioning	*n* = 8; 41.67 ± 28.17	*n* = 21; 56.35 ± 29.69	−14.68 (−39.66 to 10.30)	0.238

Values are presented as mean ± SD. Difference represents the unadjusted mean difference between children with and without additional congenital, genetic, structural, or systemic conditions (conditions present minus conditions absent), with 95% confidence intervals. Numerical two-sided nominal *p*-values are reported; Student’s *t*-test or the Mann–Whitney U test was used as appropriate. Analyses were exploratory and unadjusted, and no multivariable adjustment for potential confounders was performed. No adjustment for multiple comparisons was applied. CF was applicable only to children assessed with the Infant Scales, whereas School/Daycare Functioning was available only for children aged 2–4 years with sufficient completed items. For outcomes analysed using the Mann–Whitney U test, the reported *p*-value is rank-based, whereas the mean difference and its 95% confidence interval are provided to describe the magnitude of the between-group difference.

**Table 3:** Descriptive statistics for the PedsQL Family Impact Module.

FIM Score	Mean	SD
Total Impact Score	47.78	22.00
Parent HRQoL Summary Score	52.25	25.24
Family Functioning Summary Score	52.14	26.16
Physical Functioning	51.25	25.79
Emotional Functioning	47.83	29.26
Social Functioning	53.54	30.39
Cognitive Functioning	56.83	32.91
Communication	45.69	30.59
Worry	24.17	21.20
Daily Activities	35.00	28.57
Family Relationships	62.42	30.86

FIM, Family Impact Module; HRQoL, health-related quality of life; SD, standard deviation. Higher scores indicate better functioning.

**Table 4:** Exploratory unadjusted associations between the presence of any reported clinical condition and family-impact outcomes.

FIM Outcome	Clinical Condition Present	Clinical Condition Absent	Difference (95% CI)	*p*-Value
Total Impact Score	*n* = 49; 45.52 ± 21.77	*n* = 11; 57.83 ± 21.10	−12.31 (−26.77 to 2.16)	0.094
Parent HRQoL Summary Score	*n* = 49; 49.67 ± 25.54	*n* = 11; 63.75 ± 21.23	−14.08 (−30.68 to 2.51)	0.095
Family Functioning Summary Score	*n* = 49; median 56.25 (34.38–71.88)	*n* = 11; median 59.38 (35.94–78.13)	—	0.833

Values are presented as mean ± SD unless otherwise indicated. Differences represent unadjusted mean differences between participants with and without at least one reported clinical condition (condition present minus condition absent), with 95% confidence intervals. Family Functioning Summary Scores are presented as median (Q1–Q3) and were compared using the Mann–Whitney U test; therefore, a mean-difference confidence interval is not presented for this outcome. All analyses were exploratory and unadjusted. Numerical two-sided nominal *p*-values are reported, and no adjustment for multiple comparisons was applied. “Any reported clinical condition” denotes at least one condition reported in the caregiver-provided clinical-condition item versus none reported.

## Data Availability

The data that support the findings of this study are available from the corresponding author, upon reasonable request due to privacy.

## Supplementary Materials

The following supporting information can be downloaded at: https://www.mdpi.com/article/10.3390/jcm15197706/s1. Table S1. *p*-values for exploratory associations between demographic and clinical characteristics and PedsQL scores; Table S2. Exploratory unadjusted associations between the presence of any reported clinical condi-tion and PedsQL Family Impact Module outcomes.

## References

[1] Zuberi S.M., Wirrell E., Yozawitz E., Wilmshurst J.M., Specchio N., Riney K., Pressler R., Auvin S., Samia P., Hirsch E. (2022). ILAE classification and definition of epilepsy syndromes with onset in neonates and infants: Position statement by the ILAE Task Force on Nosology and Definitions. Epilepsia.

[2] Stafstrom C.E. (2026). Infantile epileptic spasms syndrome: Mechanisms and therapeutic approaches. Neurotherapeutics.

[3] Snyder H.E., Jain P., RamachandranNair R., Jones K.C., Whitney R. (2024). Genetic Advancements in Infantile Epileptic Spasms Syndrome and Opportunities for Precision Medicine. Genes.

[4] Feng X., Yang J., Chen N., Li S., Li T. (2025). Diagnostic yields of genetic testing and related benefits in infantile epileptic spasms syndrome: A systematic review and meta-analysis. Seizure.

[5] Nadkarni J., Jain A., Dwivedi R. (2011). Quality of life in children with epilepsy. Ann. Indian Acad. Neurol..

[6] Johansen H., Dammann B., Andresen I.L., Fagerland M.W. (2013). Health-related quality of life for children with rare diagnoses, their parents’ satisfaction with life and the association between the two. Health Qual. Life Outcomes.

[7] Wanigasinghe J., Arambepola C., Murugupillai R., Kapurubandara R., Jayawickrama A. (2021). Quality of life at 6 years in children treated for West syndrome with hormonal therapy. Epilepsy Behav..

[8] Bhanudeep S., Madaan P., Sankhyan N., Saini L., Malhi P., Suthar R., Saini A.G., Ahuja C.K., Vyas S., Singh P. (2021). Long-term epilepsy control, motor function, cognition, sleep and quality of life in children with West syndrome. Epilepsy Res..

[9] Rani A., Thomas P.T. (2019). Stress and perceived stigma among parents of children with epilepsy. Neurol. Sci..

[10] Raithatha D., Sahu J.K., Bhanudeep S., Saini L., Prinja S., Bharti B., Madaan P., Rana S.K., Negi S. (2022). Financial Burden in Families of Children with West Syndrome. Indian J. Pediatr..

[11] Kumar P., Arya V., Khanna A., Kaushik J.S. (2021). Depression, Anxiety, and Parenting Stress among Mothers of Children with West Syndrome. Indian J. Pediatr..

[12] Varni J.W., Limbers C.A., Neighbors K., Schulz K., Lieu J.E., Heffer R.W., Tuzinkiewicz K., Mangione-Smith R., Zimmerman J.J., Alonso E.M. (2011). The PedsQL™ Infant Scales: Feasibility, internal consistency reliability, and validity in healthy and ill infants. Qual. Life Res..

[13] Sato I., Soejima T., Ikeda M., Kobayashi K., Setoyama A., Kamibeppu K. (2022). Reliability and validity of the Japanese version of the Pediatric Quality of Life Inventory Infant Scales. J. Patient Rep. Outcomes.

[14] Varni J.W., Seid M., Kurtin P.S. (2001). The PedsQL 4.0: Reliability and validity of the Pediatric Quality of Life Inventory Version 4.0 Generic Core Scales in healthy and patient populations. Med. Care.

[15] Varni J.W., Burwinkle T.M., Seid M., Skarr D. (2003). The PedsQL™* 4.0 as a pediatric population health measure: Feasibility, reliability, and validity. Ambul. Pediatr..

[16] Varni J.W., Limbers C.A., Burwinkle T.M. (2007). Parent proxy report of their children’s health related quality of life: An analysis of 13,878 parents’ reliability and validity across age subgroups using the PedsQL 4.0 Generic Core Scales. Health Qual. Life Outcomes.

[17] Varni J.W., Limbers C.A., Burwinkle T.M. (2007). How young can children reliably and validly self-report their health related quality of life? An analysis of 8,591 children across age subgroups with the PedsQL 4.0 Generic Core Scales. Health Qual. Life Outcomes.

[18] Varni J.W., Sherman S.A., Burwinkle T.M., Dickinson P.E., Dixon P. (2004). The PedsQLTM Family Impact Module: Preliminary reliability and validity. Health Qual. Life Outcomes.

[19] Osborne J.P., Edwards S.W., Dietrich Alber F., Hancock E., Johnson A.L., Kennedy C.R., Likeman M., Lux A.L., Mackay M., Mallick A. (2019). The underlying etiology of infantile spasms (West syndrome): Information from the International Collaborative Infantile Spasms Study (ICISS). Epilepsia.

[20] Yao M., Ma Y., Qian R., Xia Y., Yuan C., Bai G., Mao S. (2021). Quality of life of children with spinal muscular atrophy and their caregivers from the perspective of caregivers: A Chinese cross-sectional study. Orphanet J. Rare Dis..

