# Supplementary material for: Health-Related Quality of Life and Family Impact in Young Children with Infantile Epileptic Spasms Syndrome: A Cross-Sectional Caregiver-Reported Study
Source: J Clin Med. 2026 Oct 5;15(19):7706. doi: 10.3390/jcm15197706 (PMC13666350; doi:10.3390/jcm15197706)
Supplement: Supplementary file 1 [file jcm-15-07706-s001.zip › jcm-4562666-supplementary.pdf]

Supplementary Table S1. P-values for exploratory associations between demographic and clinical characteristics and PedsQL scores.

Variable	Pooled Total Scale Score	Physical health score	Emotional Functioning	Social Functioning	Cognitive Functioning	School/Daycare Functioning
Age group, 1–24 months vs. 2–4 years	0.204	0.317	0.259	0.083	N/A	N/A
Place of residence	0.304	0.483	0.462	0.452	0.741	0.256
Parental education	0.863	0.730	0.862	0.336	0.226	0.202
Gestational age category	0.424	0.351	0.658	0.722	0.378	**0.034**
Mode of delivery	0.889	0.318	0.518	0.381	0.801	0.749
Additional congenital, genetic, structural, or systemic conditions	**0.046**	**0.012**	0.516	0.113	0.505	0.238

Values are nominal p-values from exploratory analyses and were not adjusted for multiple comparisons. Pooled PedsQL analyses were based on age-appropriate scores expressed on the 0–100 metric and should be interpreted cautiously because the age-specific instruments differ in item and domain composition. Cognitive Functioning was applicable only to children assessed with the Infant Scales (n = 17), whereas School/Daycare Functioning was calculated only for children assessed with the Generic Core Scales who had sufficient completed School/Daycare items (n = 29). Direct age-group comparisons for Cognitive Functioning and School/Daycare Functioning were therefore not applicable. N/A, not applicable.

Supplementary Table S2. Exploratory unadjusted associations between the presence of any reported clinical condition and PedsQL Family Impact Module outcomes.

Clinical characteristic	FIM outcome	Clinical condition present (n = 49)	Clinical condition absent (n = 11)	Nominal p-value
Any reported clinical condition	Total Impact Score	45.52 ± 21.77	57.83 ± 21.10	0.094
Any reported clinical condition	Parent HRQoL Summary Score	49.67 ± 25.54	63.75 ± 21.23	0.095
Any reported clinical condition	Family Functioning Summary Score	Median 56.25	Median 59.38	0.833

Values are derived from exploratory analyses and were not adjusted for multiple comparisons. "Any reported clinical condition" represents the binary variable created from the caregiver-reported clinical-condition item (at least one condition reported vs. none; present n = 49, absent n = 11). Higher FIM scores indicate better functioning. FIM, Family Impact Module; HRQoL, health-related quality of life.
